# Supplementary material for: Impact of COVID-19 on the awareness and interest in infectious disease specialization among Japanese medical students
Source: PLoS One. 2025 Sep 4;20(9):e0329451. doi: 10.1371/journal.pone.0329451 (PMC12410767; doi:10.1371/journal.pone.0329451)
Supplement: S1 Table — (DOCX) [file pone.0329451.s001.docx]

**Supplementary Table 1.**

**Breakdown list of the number of respondents from medical schools by student’s grades**

|  | Academic years | | | | | |  |
| --- | --- | --- | --- | --- | --- | --- | --- |
|  | 1^st^ | 2^nd^ | 3^rd^ | 4^th^ | 5^th^ | 6^th^ | **Total** |
| Kochi University |  |  | 103 |  | 3 |  | **106** |
| University of Miyazaki | 19 | 5 | 4 | 10 | 1 | 13 | **52** |
| Yamaguchi University | 12 | 6 | 8 | 8 | 6 | 6 | **46** |
| Hyogo College of Medicine | 21 |  | 4 | 5 | 10 |  | **40** |
| Gifu University |  | 5 | 14 | 7 | 4 | 6 | **36** |
| Yokohama City University |  | 5 | 5 | 8 | 9 | 5 | **32** |
| Shimane University | 1 | 10 | 5 | 4 | 3 | 7 | **30** |
| Kindai University | 9 | 2 | 7 | 2 | 4 | 2 | **26** |
| Okayama University | 5 |  | 5 | 3 | 5 | 2 | **20** |
| Aichi Medical University |  | 8 | 5 |  |  |  | **13** |
| Nara Medical University |  |  | 6 | 6 |  |  | **12** |
| Wakayama Medical University | 5 |  | 4 |  | 2 | 1 | **12** |
| Kyoto Prefectural University of Medicine | 7 | 1 |  | 1 |  |  | **9** |
| University of Yamanashi | 3 | 2 | 1 | 1 | 2 |  | **9** |
| Shiga University of Medical Science |  |  | 2 |  |  | 4 | **6** |
| Kobe University |  |  | 4 |  |  | 2 | **6** |
| Ehime University |  |  |  | 5 |  |  | **5** |
| Shinshu University |  | 1 | 3 |  |  | 1 | **5** |
| Osaka Metropolitan University |  |  |  |  |  | 5 | **5** |
| Tottori University |  |  | 5 |  |  |  | **5** |
| Tokushima University | 1 | 2 | 2 |  |  |  | **5** |
| Tohoku University |  |  |  |  | 3 |  | **3** |
| The University of Tokyo |  | 1 | 1 |  |  |  | **2** |
| Tohoku Medical and Pharmaceutical University |  | 1 |  |  |  | 1 | **2** |
| Kansai Medical University | 1 |  |  |  |  |  | **1** |
| Saitama Medical University |  | 1 |  |  |  |  | **1** |
| Kawasaki Medical School | 1 |  |  |  |  |  | **1** |
| Osaka University |  |  |  |  |  | 1 | **1** |
| Nippon Medical School |  |  | 1 |  |  |  | **1** |
| **Total** | **85** | **50** | **189** | **60** | **52** | **56** | **492** |
